# Reviewing Evidence for the Impact of Lion Farming in South Africa on African Wild Lion Populations

**DOI:** 10.3390/ani15152316

**Published:** 2025-08-07

**Authors:** Jennah Green, Angie Elwin, Catherine Jakins, Stephanie-Emmy Klarmann, Louise de Waal, Madeleine Pinkess, Neil D’Cruze

**Affiliations:** 1 World Animal Protection, London EC2A 4NE, UK; 2 Department of Natural Sciences, Manchester Metropolitan University, Manchester M15 6BX, UK; 3 Blood Lions, Gansbaai 7220, Western Cape, South Africa; 4 Department of Psychology, University of Johannesburg, Johannesburg 2006, Gauteng, South Africa

**Keywords:** African lion, *Panthera leo*, commercial breeding, wildlife trade, lion bone trade, lion conservation

## Abstract

The scope and scale of commercial captive lion breeding (CLB) in South Africa have rapidly increased since the 1990s. We conducted a qualitative systematic review using the PRISMA protocol to determine whether CLB provides a sustainable supply side intervention to reduce pressure on wild lion populations. A search was performed using three academic databases for sources published between 2008 and 2023. We collated and reviewed the data using an evaluation framework to determine the potential benefits and threats of CLB in the context of conservation. Among the 126 peer-reviewed and 37 grey literature articles identified, we found evidence suggesting that the framework’s criteria were not fully met, raising concerns that CLB may facilitate the demand for lions, their parts, and derivatives. Our findings further indicate a reasonable cause to doubt that the CLB provides a sustainable supply side intervention to meet the commercial demand for lions, their parts, and derivatives. This could adversely impact conservation of wild lion populations. We conclude that further research is required to effectively evaluate the purported conservation benefits of CLB. These insights may also have implications for the policy and governance of commercial predator breeding operations in South Africa and globally.

## Simple Summary

We conducted a systematic review to assess whether commercial captive lion breeding (CLB) in South Africa reduces the pressure on wild lion populations. This study reviewed the literature published between 2008 and 2023. Research unrelated to commercial breeding in South Africa was excluded, and used Google Scholar, Web of Science, and Scopus databases, along with additional sources through a snowball approach. From 126 peer-reviewed articles and 37 grey literature sources, we found insufficient data to effectively evaluate the conservation benefits of CLB. However, some studies have raised concerns that CLB may increase the demand for lions and their body parts, potentially threatening wild populations. Our review suggests that the purported beneficial impact of CLB practices on wild lion populations requires caution and may not offer a sustainable supply side solution that meets commercial demand due to the potential negative impacts on wild lions. These findings underscore the importance of further research and have potential implications for the regulation and governance of predator breeding operations in South Africa and beyond.

## 1. Introduction

Commercial captive breeding of wild species, also known as wildlife farming, is often perceived as an effective supply side approach to meet the demand for wildlife commodities while reducing the pressure on wild populations [1,2]. Breeding a captive supply of wildlife can reduce wild-harvesting pressure by saturating the market with affordable products from farms [3,4]. However, alternative views warn that such interventions may be counterproductive because they can potentially increase pressure on wild populations by encouraging the demand for wildlife products, such as bones, teeth, and claws. Furthermore, it can increase opportunities for laundering poached wildlife through legal commercial trade and may require taking animals from the wild to replenish captive stock, improve genetic diversity, and/or breed for specific traits [5,6,7,8,9].

Considering the mixed evidence on this topic, only a handful of frameworks have been established to assess the impact of wildlife farming on wild populations [1,5,10,11,12]. However, most of these approaches are limited by complex market conditions that depend on the unpredictable responses of commodity traders [6]. Nevertheless, evaluating the impact of commercial wildlife farming on a per-species and market-specific context is imperative to reduce the likelihood that captive wildlife farms adversely affect wild populations [3,12].

In South Africa, an estimated 7838 African lions (*Panthera leo*) are farmed across 342 commercial facilities to meet the demand for lions as commodities [13]. The commercial use and trade of African lion body parts and derivatives are well documented in South Africa and internationally [14]. Large quantities of lion skeletons have been exported to East and Southeast Asia to meet the market demand for big cat bones as an ingredient in traditional Chinese medicine (TCM) [15,16]. As early as 2005, lion bones were found as an ingredient in “bone strengthening wine” in China and have since been widely used as a substitute and supplement to tiger (*Panthera tigris*) bones [17]. However, since 2019, the legal lion bone trade has been halted in the absence of a Convention on International Trade in Endangered Species of Wild Fauna and Flora (CITES) annual export quota following the High Court lion bone judgement [18]. Additionally, lion trophies, such as head mounts and skins, are exported internationally by recreational trophy hunters [19], and live lions are exported to Southeast Asia for zoos, breeding facilities, and private collections [13]. Live lions are traded domestically for trophy hunting, breeding to slaughter for bones, parts, and derivatives, and for tourism, where volunteers and tourists pay for hands-on experiences with lions [20]. Lion skins, claws, teeth, and bones are also used for traditional and cultural purposes across lion range states [14,21] alongside other body parts such as tails, reproductive parts and internal organs that are also reportedly harvested [22]. Most of the international commercial trade of lion parts to Southeast Asia and the majority of domestic trade in live lions is sourced from the captive population in South Africa [15], whereas local and regional uses of lions for traditional and cultural purposes usually come from wild populations across their range states, although some captive-bred sourcing has been reported [14].

African lions are listed as Vulnerable and decreasing on the International Union for Conservation of Nature (IUCN) Red List of Endangered Species; however, in South Africa, they are listed as Least Concern, mainly due to managed populations in fenced private reserves [23]. Wild lion populations in South Africa are intensively managed and remain stable [24], existing primarily in a few large national parks and other protected areas along with a significant number of smaller managed reserves [25]. The main threats to lion populations include habitat loss, prey depletion, and human-lion conflict; however, targeted poaching for their body parts was noted as an emerging threat in the most recent IUCN species assessment [22]. Furthermore, there is increasing concern over the impact of illegal lion trade across range states, as well as increasing numbers of targeted poaching incidents, which are suspected to be related to domestic, regional, and international trade in body parts [14,26,27,28]. The South African Scientific Authority reportedly considered the impact of lion farming on wild populations in South Africa to be minimal [29], and previous studies have concluded that there is little evidence of increased poaching of wild lions to supply this trade [15,30]. However, the lack of conservation value of commercial lion farming has been noted, and the role of farms in stimulating illegal wild lion offtake remains ambiguous [31,32,33]. The overall impact of the trade in lions and their parts on wild populations remains largely unquantified [34].

Commercial captive breeding of wildlife may have both positive and negative impacts on the conservation status of wild populations [35]. In this study, we conducted a qualitative systematic review of the available literature on commercial lion farming in South Africa. The aim of this study is to assess the available evidence on how South Africa’s commercial lion breeding (CLB) industry affects the conservation status of wild lion populations in Africa using a pre-existing evaluation framework by Tensen [1]. This may answer the question of whether captive breeding as a tool to meet the demand for lion commodities reduces the pressure on African wild lion populations.

## 2. Materials and Methods

### 2.1. Search Strategy

We conducted a systematic literature review to identify appropriate sources for reviewing the evidence currently available on the impact of commercial captive breeding of lions in South Africa on the conservation status of wild lion populations. This systematic review was conducted following the “preferred reporting items for systematic review and meta-analyses” (PRISMA) recommendations [36]. The PRISMA checklist is presented in Appendix A.1. This systematic review was not formally registered, and the review protocol has not been published; however, further details can be made available upon request from the corresponding author.

In August 2023, we searched three academic databases (Google Scholar, Web of Science, and Scopus) using 20 search terms. Searches used a combination of the terms “commercial lion farming” and “commercial lion breeding” with the Boolean operator AND, followed by the terms “South Africa”, “traditional medicine”, “bone trade”, “skeleton trade”, “poaching”, “trafficking”, “laundering”, “consumer demand”, and “wild populations”, plus three additional searches for “commercial captive lion breeding South Africa”, “*Panthera leo* AND commercial farming”, and “*Panthera leo* AND commercial captive breeding”. To be included, an article had to be in English, related to the commercial farming of lions in South Africa, and published between 2008 and August 2023. We limited the search to articles published between these years to capture all relevant information starting from the first year that exports of captive-bred lion skeletons from South Africa were legally traded and recorded by the CITES. The full texts of all potentially eligible studies were retrieved and independently screened for inclusion by a team of five researchers (JG, CJ, LdW, SEK, and MP). We excluded articles that focused on lion breeding for research purposes, lions housed in zoos, lions bred outside South Africa, and the commercial farming of other large felids. Studies that did not meet the inclusion criteria were excluded, and the reasons for exclusion were recorded during full-text screening. All other peer-reviewed articles returned in the search were included.

In addition, we conducted a ‘snowball’ search in August 2023 using the reference lists of all eligible peer-reviewed articles to identify further appropriate articles and reports. The inclusion of grey literature was particularly important for identifying underreported aspects or findings outside of traditional academic publishing, thus mitigating publication bias. The full texts of all potentially eligible articles and reports were retrieved, and the same team of five researchers (JG, CJ, LdW, SEK, and MP) independently screened the full texts for inclusion. Here, we included grey literature sources, such as government documents, media articles, and reports from relevant conservation, animal welfare, and environmental organisations or stakeholders, within the same inclusion criteria outlined for the peer-reviewed datasets.

### 2.2. Review Framework

We conducted a qualitative systematic review of all sources identified in the literature search to extract relevant information that could serve as evidence of the impact of CLB on the conservation status of wild lion populations. We reviewed the articles in our dataset against criteria adapted from a pre-existing evaluation framework by Tensen [1], similar to the study by Davies et al. [35]. This framework was designed to systematically assess the quality and relevance of evidence concerning the impact of CLB on wild lion populations. The criteria in the framework cover a broad range of potential impacts of commercial wildlife farming on wild populations and provide a basis for objectively determining the potential benefits and threats of lion farming in a conservation context. However, we also view the framework as a tool for identifying areas where knowledge is lacking and where greater caution or research into the potential impacts on wild populations may be necessary (Davies et al. [35]).

A data extraction Excel spreadsheet was developed, pilot-tested on five randomly selected articles, and subsequently refined. A team of seven researchers (JG, AE, CJ, SEK, LdW, NDC, MP) independently evaluated each eligible article against the criteria in the framework. Criterion 1 relates to consumer preferences, criterion 2 relates to information on the market supply and demand for lion products, criterion 3 relates to the cost efficiency of lion farms, criterion 4 relates to breeding stock, and criterion 5 relates to information on criminal activity in the CLB industry [1] (Table 1). Each article in the dataset was examined by one of the seven reviewers, who searched the article in its entirety and recorded any text in support or contrary to these criteria. Applying these structured, predefined criteria consistently across all sources reduced the risk of selective reporting. The coding process applied by the reviewers was a binary “yes” or “no” response for whether each article contained text relating to criteria 1–5, as well as whether it contained text relating to wild lion populations in other range states to capture potential threats to wild lion populations outside South Africa. The corresponding text for each criterion was extracted into an Excel spreadsheet, and one researcher (JG) checked all extracted texts against the conditions set out by the framework. Inconsistencies were discussed by the team of seven researchers until a consensus was reached. The data type was also recorded, where primary data were defined as original evidence based on the analysis of data, secondary data were defined as the presentation of evidence from other sources, and anecdotal data were defined as observations and expert knowledge, for example, personal communications or observations reported by the authors in the field. Finally, all extracted eligible texts were summarised by one researcher (JG) according to the conditions set out by the framework. Each of the seven researchers then reviewed the summarised text against the articles that they independently reviewed to check for missing or misinterpreted data, and the data were corrected accordingly. We present peer-reviewed and grey literature sources as two separate datasets in our results and discussion. We did not consider grey literature as primary data and presented it purely as supporting information, as we recognised that grey literature sources may present information with specific agendas or without peer review. However, we used the same review framework for both the assessments.

## 3. Results

**Figure 1:**
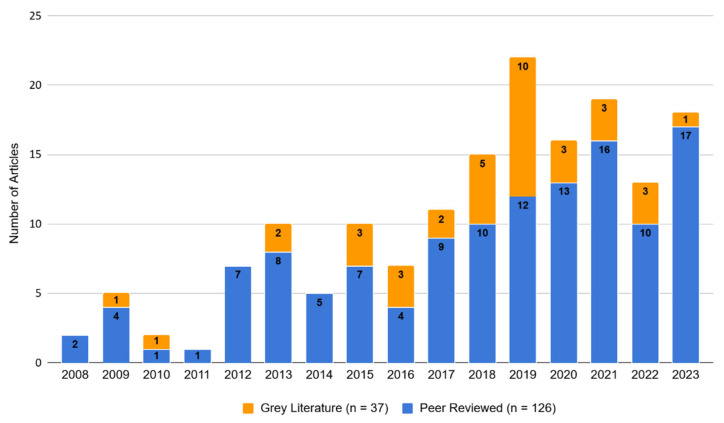
Number of articles per year of publication categorised by peer-reviewed (*n* = 126) and grey literature (*n* = 37) sources for 2008–2023. All sources are detailed in the database in Table A2 and Table A3.

Our initial literature search returned 152 articles. We excluded five articles published outside our specified date range and a further 10 that we were unable to access via academic institutional logins or open-access hosts. The remaining dataset comprised 125 peer-reviewed literature sources (113 journal articles and 12 book chapters) and 12 grey literature sources (four postgraduate theses, three non-peer-reviewed journal articles, three institutional reports, one media article, and one blog post). Our snowball search yielded one peer-reviewed IUCN assessment and 25 grey literature sources (comprising five media articles, two National Assembly questions, one court case summary, three government reports, one postgraduate thesis, one blog, 12 institutional reports, and one peer-reviewed IUCN assessment). All sources are listed in Appendix A.2. Figure 1 shows the number of articles of each source type published over the study period of 2008–August 2023.

**Figure 2:**
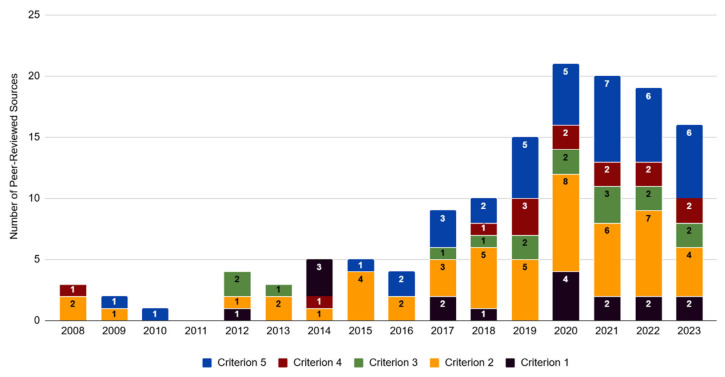
Number of times each predetermined criterion was noted in peer-reviewed sources indexed per year of publication for 2008–2023 (*n* = 70 out of a total of 126 articles). The framework used for evaluating the species conservation impacts of commercial lion farming was adapted from Tensen [1]. Criterion 1 relates to consumer preferences, criterion 2 provides information related to the market supply and demand for lion products, criterion 3 relates to the cost efficiency of lion farms, criterion 4 relates to breeding stock, and criterion 5 provides information relating to criminal activity in the CLB industry.

Across the peer-reviewed literature dataset (*n* = 126), 56% (*n* = 70) of the articles contained text relating to at least one of the five predetermined criteria outlined in Table 1 (Figure 2). The highest number of articles was recorded for criterion 2, information related to the market supply and demand for lion products (40%, *n* = 51; Table 2), followed by criterion 5, information related to criminal activity in the industry (33%, *n* = 42). A smaller number of articles contained information for criterion 1, relating to consumer preferences (11%, *n* = 14), criterion 3, relating to the cost efficiency of farms (13%, *n* = 16), and criterion 4, relating to breeding stock (11%, *n* = 14). The majority of peer-reviewed articles in our dataset (70%, *n* = 87) were published since 2017, which may be related to the fact that South Africa’s CLB industry came under increasing scrutiny as a result of cases of animal cruelty and neglect reaching the public domain, as well as the unregulated nature of the industry, e.g., [37,38,39].

**Figure 3:**
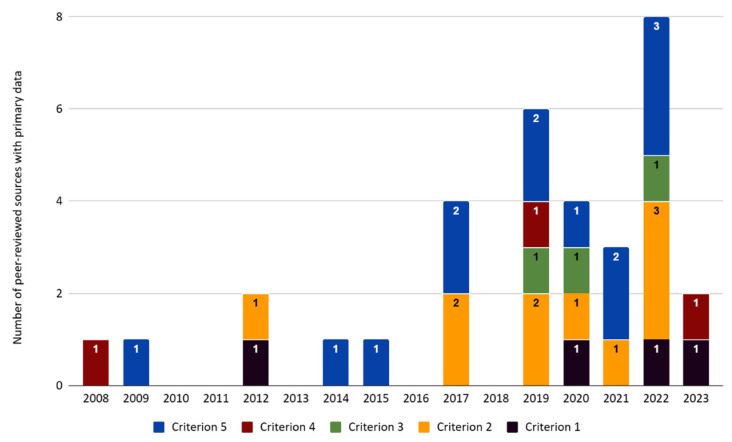
Number of times each predetermined criterion was noted in peer-reviewed sources presenting primary data on each predetermined criterion for 2008–2023. The framework used for evaluating the species conservation impacts of commercial lion farming was adapted from Tensen [1]. Criterion 1 relates to consumer preferences, criterion 2 relates to information related to the market supply and demand for lion products, criterion 3 relates to the cost efficiency of lion farms, criterion 4 relates to breeding stock, and criterion 5 relates to information relating to criminal activity in the CLB industry.

Of the articles that were recorded as containing text related to the five criteria, the majority contained secondary information as context or discussion. A much smaller proportion of the dataset presented primary data or anecdotal data containing new information as evidence to support or oppose the criteria. Four articles presented primary data relating to criterion 1 (29% of 14 articles for this criterion), 10 for criterion 2 (20% of 51 articles), three for criterion 3 (19% of 16 articles), three for criterion 4 (out of *n* = 14, 21%), and 13 for criterion 5 (31% of 42 articles) (Figure 3).

**Figure 4:**
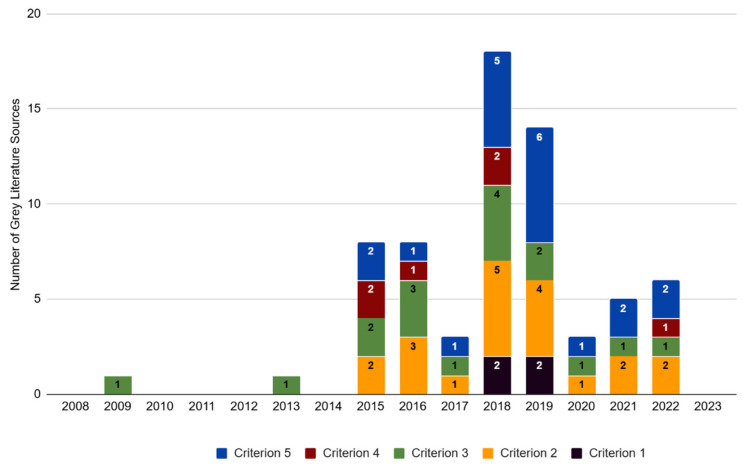
Number of times each predetermined criterion was noted in grey literature sources indexed per year of publication for 2008–2023. The framework used for evaluating the species conservation impacts of commercial lion farming was adapted from Tensen [1]. Criterion 1 relates to consumer preferences, criterion 2 relates to information related to the market supply and demand for lion products, criterion 3 relates to the cost efficiency of lion farms, criterion 4 relates to breeding stock, and criterion 5 relates to information relating to criminal activity in the CLB industry.

Across the grey literature dataset (*n* = 37), 73% (*n* = 27) of the articles contained text relating to at least one of the five criteria in Table 1. The highest number of articles were recorded for criteria 2, 3, and 5 (54%, *n* = 20; 46%, *n* = 17; and 54%, *n* = 20, respectively) (Figure 4). A smaller number of articles contained text relating to criterion 1 (11%, *n* = 4) or criterion 4 (16%, *n* = 6). Again, for the grey literature dataset, the majority were published since 2017 (73%, *n* = 27).

Across the full dataset (*n* = 163), 55 articles (34%) contained information relating to lion populations in range states outside South Africa, comprising 44 articles (27%) from the peer-reviewed dataset (*n* = 126) and 11 articles (30%) from the grey literature dataset (*n* = 37).

## 4. Discussion

Below, we interrogate the peer-reviewed and grey literature sources identified through our searches in more detail for the five predetermined criteria of the evaluation framework adapted from Tensen [1] (Table 1 and Table 2).

### 4.1. Criterion 1—Legally Farmed Lion Parts Provide a Substitute for Wild Lion Parts

Criterion 1 describes whether legally farmed lion parts could provide a genuine substitute for wild lion parts in terms of consumer preferences. For farmed lion parts and derivatives to be a suitable alternative to wild lion parts, there should be no noticeable preference difference for consumers.

#### 4.1.1. Evidence Sourced from Peer-Reviewed Literature

In the context of lion bone-based traditional medicine products, one quantitative study assessing preferences among the general public in China and Vietnam found that there was no overriding preference for wild or farmed sources in either country [40]. Two articles noted that the concept of a binary distinction between wild and captive sources may be overly simplistic from the consumers’ perspective, and the combination of different large felid products of different origins on offer complicates the investigation of product preferences [14,55]. Data from the perspective of wildlife traders and traditional health practitioners (THPs) also indicated a mix of preferences for either captive or wild-sourced lions [14]. Among THPs, 25% of respondents stated a preference for using body parts from wild lions over farmed ones. Strength and purity were cited as reasons for using wild-sourced lions, whereas background information about the lion’s age, diet, and medical history was cited as a reason for preferring captive lions.

In the context of trophy hunting, there are again mixed preferences for captive versus wild lions. For example, it is important to hunters that the lions they kill for their trophies are aesthetically pleasing to be taxidermied and mounted [41]. Thus, captive-bred lions are preferred for their selectively bred distinctive characteristics, such as large dark manes, and are typically in better condition than wild lions [20,56]. Captive lion hunts are also appealing to certain hunters because they are virtually guaranteed to result in a kill and often yield larger trophies than wild lions [42,56]. Hunting packages for captive lions can attract a wider range of customers because they can be delivered in a much shorter time frame than for their wild counterparts and are thus much cheaper and cater to tourists who have less time and/or money available [56]. Captive hunts are also more accessible to tourists with physical limitations or lack of experience [42,56]. Some hunters also show a preference for captive lions for the perceived benefit of conserving wild populations [41].

However, farmed lions are not unanimously considered a viable substitute for wild lions. Data pertaining to preferences among hunters collected in 2012 reported that 76% of clients interviewed would prefer their next lion hunt to be for a wild lion, in comparison to 17% that would prefer a captive-bred lion and 7% that had no preference [42]. Some members of the hunting community consider captive-bred lions less “authentic” than wild lions and enable unskilled hunters to participate [41,56].

#### 4.1.2. Evidence Sourced from Grey Literature

The grey literature added little further evidence to what has already been described in Section 4.1.1. One article noted that consumers in Southeast Asia are allegedly prepared to pay more for bones from wild lions due to the belief that their effects are more potent than those of captive lions, but this was not substantiated with data [57]. Another article reported that traditional medicine consumers in South Africa sometimes purchase fake lion fat due to lack of awareness that the product is inauthentic [58].

#### 4.1.3. Summary

Our results show that there has been limited insight into consumer preferences for lion bone products, and many consumers may not be aware that they are purchasing lion products at all. The available evidence indicates that most lion bone traditional medicine consumers and traders show no preference for captive or wild sources, although in one study, farmed lion bone was chosen as the least preferred option among survey participants, indicating that farmed lions may not be an adequate substitute [40]. It was acknowledged that the results may differ if the survey was exclusively conducted among actual consumers and that more research is needed in this regard [40]. There was also recognition that reported preferences do not always reflect the actual preferences shown in consumption patterns [40,59]. The available evidence among hunters indicates mixed preferences for wild and captive-sourced lions; thus, captive-bred lions cannot be broadly considered a substitute for wild lion hunters [42]. Thus, we surmise that criterion 1 cannot be fully satisfied due to ambiguity and mixed preferences across the evidence base. Furthermore, research gaps exist pertaining to preferences among actual consumers rather than the general public in consumer countries.

### 4.2. Criterion 2—Farmed Supply of Lion Parts and Derivatives Serves Current Market Demand

For criterion 2 to be met, the production of captive lion products must be sufficient to supply the existing demand without creating additional demand. We specifically focused on information relating to changes in the supply and demand for, and traded quantity of, lion parts and derivatives, as well as evidence of new markets emerging for lion parts in relation to the farming industry.

#### 4.2.1. Evidence Sourced from Peer-Reviewed Literature

The market supply and demand for lions and their parts and derivatives has fluctuated and evolved since 2008, with an overall exponential increase up to 2019, when no further annual CITES export quota was set by the South African government. The international commercial trade in lion parts started with the export of trophies from the hunting industry, but since 2008, CLB has proliferated to supply lion bones for export to Southeast Asia for use in traditional medicine products. The majority (65%) of commercial captive lion facilities in South Africa surveyed by Williams and ‘t Sas-Rolfes [19] supplied lions for both trophy hunting and bone trade purposes, but many facilities reportedly increased their focus on the lion bone trade following the United States ban on trophy imports in 2016 [15]. With the expansion of the CLB industry, the use of lion cubs and young adults as interactive tourist attractions has also emerged as a new market, creating additional demand for breeding and trading lions for revenue generation [31,43,60].

Initially, lion bone was thought to supplement or substitute tiger bone in TCM products [61], but recent evidence across our dataset suggests an emerging demand for lion parts, specifically in Vietnam, and to a lesser extent, China [28]. Market prices can be a valuable indication of market trends, and the available data show that the wholesale value of a lion skeleton more than doubled from 2012 to 2019 [19]. Such rising prices can indicate a widening gap between supply and demand, suggesting an increasing demand for skeletons during this period.

From a supply perspective, the available data indicate that the majority of the demand for international trade in lion parts and derivatives from South Africa is met by captive supply instead of wild-sourced lions [15,42,56]. The supply of captive lion parts may be limited by imposed restrictions. For example, of the 23 CLB facilities that sold bones in 2016, 20 said they could supply a larger quantity of bones, and two more said they could supply the same quantities, if restrictions did not apply [19].

In addition to the international trade in lion bones, there is also a pan-African market for lion bones for traditional medicinal and spiritual purposes [21]. THPs in South Africa use lion parts [44], and some traders have reported increasing requests for lion parts due to changing medicinal practices and foreign influence [14]. From the available data, the role of lion farms does not seem to influence the demand for traditional belief uses, and farmed wild animals lack the symbolic value compared to wild-caught products, rendering it less likely that farmed parts will meet this demand [62]. Local conservation professionals have raised concerns that some rural Africans are increasingly motivated to supply markets with body parts from poached wild lions [63]. Southern African wild-sourced lions are more likely to be used for local cultural and medicinal purposes, as evidenced by respondents from interviews with THPs and traders who indicated that lion parts were obtained from a variety of sources, including wild lion populations and occasionally breeding farms [14].

Although limited, there is some evidence that captive lion hunts may serve current market demands, thereby having a positive impact on the conservation of wild lions. For example, the number of wild lion trophies exported from African countries declined by nearly a third between 1994 and 2010, which may have been due, in part, to the increased availability of captive-bred lion hunting options in South Africa [64]. It was also estimated that hunting captive lions in South Africa could keep more than 1000 lion hunters away from hunting wild lions in other parts of Africa [65], and some speculate that captive-bred lions may reduce the pressure from hunters for wild lions [42]. However, much of this information is speculative without substantive data to support it, and many other factors can contribute to fluctuations in demand.

#### 4.2.2. Evidence Sourced from Grey Literature

The supply of captive-bred lions has evolved with changing markets. Two sources [58,66] reported that selling bones internationally was a secondary benefit of the hunting industry, suggesting a lack of economic incentive to farm lions solely for their bones due to the associated costs. In contrast, one peer-reviewed article reported that 30% of commercial captive facilities breed and keep lions purely for trade in their bones and derivatives [19]. However, a grey literature source noted that the vast majority (91%) of skeletons exported since 2017 had intact skulls, indicating that they were not a by-product of captive trophy hunting but were bred specifically for their bones [38]. One article [57] suggested that if the demand for lion bones in the East Asian trade continues to grow, bones from the captive hunting industry are unlikely to satisfy that demand, particularly as the demand for captive hunting has reduced considerably since the US trophy import ban in 2016 [57].

It has also been reported that lion farming in South Africa may be stimulating the market supply for the intensive commercial breeding of lions in other geographic regions, and that an increase in farming can, in turn, influence demand. An NGO report [67] noted that Pakistan imported 139 live lions between 2011 and 2020 and had 20 farms specialising in breeding non-native big cats in one province alone. Lion farms are already believed to exist in Vietnam and are predicted to increase in other consumer countries, possibly supported by the information that South Africa exports live captive-bred lions to Bangladesh and China [38].

Despite the lack of history of lion bone use in traditional medicine in East and Southeast Asia [61], there is an existing demand for lion bones [58,68], and they are now permitted as an ingredient in medicinal wines in China, which traditionally contained tiger bones. It is unclear whether consumers are aware that these products contain lion bone as a substitute, as it has been reported that significant effort is made to market lion bone products by implying that they contain tiger bone [68]. However, it is also noted that there is evidence of Chinese nationals purchasing lion products in neighbouring countries to take home, and an online survey found a variety of lion claw and teeth products for sale online in China [68]. Similarly, in Vietnam, lion bone is processed into cake and balm products, claws and teeth are worn, and skulls are displayed as status symbols [38]. These products are indistinguishable from tigers, and only a few products advertised as being made from lions were observed during surveys in Vietnam [68]. However, anecdotal evidence suggests an increasing demand for lion products [38,68]. One stakeholder reported that sellers are now openly telling consumers that the cake contains lion, and consumers are specifically requesting lion products [38], often advertised as coming from South Africa [68]. One source suggested that the demand for lion products is high enough that the market contains fake plastic versions [68].

#### 4.2.3. Summary

There are three distinct but related supply trade chains in the market for lion bones [15], and evidence across our dataset indicates that lion farming is making a notable contribution to the global trade of lion parts. Some commercial uses for lions have emerged as a direct result of captive breeding, for example interactive tourism experiences with lions, providing another revenue stream for captive facilities. However, it is unclear whether the demand for lion bones in Southeast Asia is met by the captive population in South Africa or stimulated by it. Some stakeholders noted that there could be an unquantifiable demand for lion products due to growing consumer numbers and wealth in Asia [45], which could put further pressure on wild populations. We conclude that there is currently insufficient evidence available to determine whether the supply of captive lions in South Africa satisfies the international demand for lion body parts in East and South-East Asia; therefore, this criterion is violated.

### 4.3. Criterion 3—Lion Parts and Derivatives from Farmed Populations Are More Cost-Efficient than Wild Counterparts

To satisfy criterion 3, lion trophies, bones, parts, and derivatives from captive-bred populations need to be equally or more cost-efficient than their wild counterparts.

#### 4.3.1. Evidence Sourced from Peer-Reviewed Literature

Of the 15 articles that provided relevant information for this criterion, only one provided a direct comparison of the price points between wild and captive lion hunts [42]. However, in terms of lion products, it was noted that skeletons from captive-origin lions were considered cheaper alternatives to other large felids [15,42] and that captive hunts are typically cheaper than wild hunts [56], undermining the financial value of wild lion trophies [69]. Furthermore, human-reared cubs may be financially more valuable in adulthood than their wild counterparts [20], but no specific comparative data on the relative prices in each market were available. Increasing profitability on breeding farms is sometimes achieved by practices that compromise the health and welfare of mothers and cubs, such as speed breeding [20], whereby breeders remove young cubs from lionesses before they are weaned to force a premature return to oestrus for faster breeding cycles and increased revenue per lion [60]. No information is available on the comparative revenue that would be generated if facilities were limited to natural breeding cycles. In addition, information about the cost of maintaining CLB facilities and the comparative cost of ensuring that facilities adhere to animal welfare standards and legislation is missing from the articles in our dataset. There could be a considerable difference in profit margins between the current standards in commercial captive facilities and the profits generated if higher welfare standards were adhered to in commercial captive facilities.

The range of revenue streams generated from lions and their derivatives from commercial captive facilities can increase the cost efficiency of farmed populations through means that are not possible for their wild counterparts. For example, selective breeding for specific traits that are rare in wild populations, like white lions, can fetch higher prices than the more common tawny lion trophies, sometimes up to five times the price [56]. Some commercial captive lion facilities also boost profitability by diversifying income streams, such as hosting paying international volunteers to tend to captive lions, which can generate up to US$98,000 per month of additional income for a single facility [56]. Diverse income streams can provide profit stability in fluctuating markets. For example, when the number of captive trophy hunts fell by nearly one-third after the US trophy import ban in 2016, farms were kept afloat mostly by the sale of lion skeletons to Asia [70].

Wild lion parts are considered very profitable among THPs in South Africa [44], and high prices charged by traders to THPs for lion parts in urban areas have been anecdotally reported [19]. Despite this, the relative price of wild and captive lion products and the absolute contribution of lion sales to THPs’ income have not been assessed [14,44].

#### 4.3.2. Evidence Sourced from Grey Literature

Several sources have provided information on price points for lion parts along the trade chain, such as the typical operating costs of breeding farms, the purchase price of lion parts in Southeast Asia, and the costs associated with captive hunting [38,71,72]. However, no comparative data between wild and captive sources have been presented, and the overall profitability of parallel industries has not been quantified. A recent court challenge highlighted the profitability of lion bones, with the value of a full, intact male skeleton estimated at US$3600 and a female skeleton at US$3100 for South African captive breeders [73].

#### 4.3.3. Summary

It was noted that obtaining accurate price data is challenging [19], and overall, very little is known about the economic details along the trade chains relating to lions, and the cost-effectiveness of captive breeding has yet to be demonstrated [38,57]. Although no definitive evidence exists that captive-bred lions can be produced less expensively than poaching lions from the wild [57], it is evident from our dataset that commercial captive facilities can generate additional revenue from diversified income streams. No direct comparison of price points or overall profitability between wild and captive sourced lion products is available; therefore, criterion 3 is not fully satisfied.

### 4.4. Criterion 4—Captive Populations Can Be Maintained Without Restocking from Wild Populations

To satisfy criterion 4, captive populations must be maintained with sufficient variation without restocking from the wild population.

#### 4.4.1. Evidence Sourced from Peer-Reviewed Literature

There is some evidence from survey responses and further anecdotal data indicating that a small number of lions have been sourced from wild populations outside of South Africa, such as for aesthetic purposes to introduce darker manes to the captive population [47]. However, the available information suggests that most of the captive population was sourced in South Africa from other breeders [19,45,47]. According to data from surveys with lion facility owners, captive-origin lions from South African breeders were acquired by 87% of the respondents to stock their facilities when they opened, with only a small number (5%) sourcing wild lions from reserves in South Africa and Botswana as founder stock [19]. Industry stakeholders interviewed by researchers reported that it is standard practice to swap lions between commercial captive facilities with different bloodlines to sustain the captive population while preventing inbreeding [48]. However, caution is required [57] when relying on interview data, which lack audited financial statements and clarity regarding the parameters of a healthy captive population. This raises doubts regarding the claims made by van der Merwe et al. [74] that breeding creates healthy captive populations. This is supported by previous concerns regarding health issues caused by inbreeding in the captive population [39]. Several articles have highlighted the lack of transparent information regarding breeding practices and the need for a reliably maintained studbook [60,75], which would strengthen the evidence for this criterion.

Although strictly outside of criterion 4, Booyens [76] showed that rewilding of captive-bred lions is plausible in small reserves with a limited number of other large predators. However, the use of captive-bred lions for rewilding purposes is currently not a conservation requirement in South Africa, as there is a surplus of metapopulation wild lions that can be used for restoration purposes, even beyond South Africa’s borders [13].

#### 4.4.2. Evidence Sourced from Grey Literature

No further primary evidence regarding the maintenance of captive lion populations was presented in the grey literature dataset.

#### 4.4.3. Summary

There is limited information relating to captive lion stock in the literature in our dataset. The genetic background of the captive lion population in South Africa is mostly unknown due to a lack of official records [77,78], and no peer-reviewed studies have presented data to demonstrate that the captive population is responsible for maintaining genetic variation [43]. While a lack of supporting evidence adds uncertainty, there is very limited evidence to suggest that captive populations are being restocked from wild lion populations. The South African Predator Association (SAPA) guidelines state that breeding animals should not be sourced from wild populations [56], and the data we found across peer-reviewed and grey literature sources suggested that the captive population is maintained by trading breeding lionesses between commercial captive facilities [48]. However, there are knowledge gaps that disable criterion 4 from being fully satisfied, but the available literature indicates that there is currently no cause for concern that wild lions are taken to stock captive facilities [19,47,48].

### 4.5. Criterion 5—Wild Populations Are Sufficiently Protected from Criminal Activity Relating to Lion Farm Facilities

To determine evidence for criterion 5, we reviewed our dataset for information related to the laundering and poaching of wild lion parts and derivatives, techniques for distinguishing between captive-bred and wild-sourced lion parts, and evidence relating to enforcement capacity and regulatory oversight.

#### 4.5.1. Evidence Sourced from Peer-Reviewed Literature

Interviews with THPs and traders revealed that a number of respondents sourced lion parts from poachers and that some captive lion breeders donated or sold lion parts to THPs [14]. One source reported anecdotal evidence from a Senior Superintendent in the South African Police Services Forensic Laboratory who believed that poaching of lions to supply the Asian market was occurring in South Africa [49]. Similarly, another source reported anecdotal evidence that may flag the presence of illegal lion trade in South Africa and noted a rise in incidents of lion poisoning on private property in the country since 2015 [21]. Survey respondents reported anecdotal evidence of wild lion carcasses smuggled into South Africa from Zimbabwe for illegal trade, potentially indicating the laundering of wild lions from other range states through South African captive facilities [21,45]. Anomalous CITES permit declarations have also been detected multiple times, alongside serious errors by issuing authorities on export permits [15]. Illicit trade in lion parts by organised criminal syndicates has also been reported [15].

It was identified that the CLB industry is governed by a patchwork of contrasting legislation across multiple provincial and national authorities, with disparities that leave legal loopholes [50]. Although systems have been implemented to prevent laundering attempts through a verified chain of custody process [16,51], irregularities and compliance errors have been detected [46]. There are also some illicit behaviours that would not be captured in the CITES compliance procedure because they occur prior to lions leaving facilities [46]. The data also showed limitations in regulatory oversight stemming from a lack of adequate record systems, rendering fraud difficult to monitor [46].

Sources across our dataset noted increasing incidences of poaching of lions as well as evidence of domestic, regional, and international trade in lion parts from illegal sources due to their proliferating value [22,47,57,68,79]. It has been suggested that the high prices charged for farmed lion skeletons in South Africa may lead to the development of a parallel market, whereby poached wild lions will be supplied to one segment of the market and farmed lions to another [38]. New insights were provided into the procedures and mechanics of how some lion facilities use legal activities, such as commercial captive breeding and hunting, to feed into the illegal international lion bone trade [80]. However, it was also noted that the reportedly high revenue for bone farmers is misleading when accounting for the costs subtracted by intermediaries along the supply chain, and the actual revenue generated by owners of commercial captive facilities is substantially less than suggested, reducing the likelihood that illegal hunting is incentivised by high prices from captive sources [58].

With regards to distinguishing between captive-bred and wild-sourced lions, one source presented data from cranial and mandibular morphology analysis showing that captive lions tend to have smaller skulls than wild lions [52]. However, this approach is likely impractical for distinguishing between traded specimens due to the need for intact specimens and the capacity to distinguish between species or possible hybrids [53]. These researchers also tested morphological differences between the skulls of tigers and lions and provided a guide to the average mass of lion skeletons and skulls [53]. They concluded that while it is possible to use morphological differences to identify illegal activity, this is not a foolproof method for distinguishing between species. The mass of consignments of lion bones could be used to inform predictions of the accuracy of a declaration on a CITES permit if the parts are not intact [53]. However, weaknesses were identified in this approach that allowed for undetectable fraudulence due to year-on-year differences in the mean skeleton weight from 2016 onwards [16]. Another study attempted to differentiate between captive-bred and wild lion hair through carbon and nitrogen stable isotope analysis [54], which may have potential applications to lion bone samples, but the analyses were inconclusive [51]. Finally, DART mass spectrometry was used for the chemical distinction between bone types [51]. This approach was able to differentiate between batches of captive-bred South African lion bones and batches of wild lion bones, but further testing is needed to confirm the suitability of this technique and the resources required. No genetic test can currently distinguish captive-bred lions from wild lions [49].

#### 4.5.2. Evidence Sourced from Grey Literature

There is a plethora of discussions about illegal activities relating to lion farming and lion bone trade across the grey literature, much of which echoes the evidence described in the peer-reviewed literature (Section 4.5.1) [22,38,57,58,67,68,79,81]. There is some evidence of a link between the CLB industry and poaching of wild lions, as indicated by a 2012 court case against a wildlife trafficking syndicate that revealed that contact with the criminal network was initiated by members of the South African captive predator industry [38]. Although not strictly laundering, another investigation report revealed that some CLB facilities use legal activities like hunting, to hide their involvement in the illegal bone trade, using concealment tactics to avoid detection during inspection, although sources did not reveal any direct connection to a specific criminal network [81]. An investigation in Southeast Asia determined that the addresses and destinations for skeletons legally exported from South African farms could not be verified or were not known, indicating criminal involvement related to sanctioned CITES permits [38].

#### 4.5.3. Summary

Evidence for criterion 5 indicates inadequate protection from criminal activity. Much of the text relating to the impact of lion farming in South Africa on poaching of wild lion populations is speculative. One source suggested that the willingness of South African lion breeders to supply lion body parts could put increased pressure on wild stocks elsewhere, but did not provide data to substantiate this [56]. It was noted that survey responses from lion bone traders in South Africa regarding the potential impacts in other countries appeared to be based on suspicion and speculation, requiring further verification or corroborating evidence [15]. Most of the available data are sporadic and anecdotal; formal investigation of the causal effects of commercial lion farming on the status of wild populations has often been overlooked, and thus the impact is largely undocumented [15]. Although direct links between lion farming and declines in wild populations elsewhere have yet to be evidenced, there is concern that the situation has arisen or could arise [26,28]. A further concern is that the 23 lion range states that are parties to CITES have been assessed as having inadequate legislation for the implementation of CITES regulations [82], failing to protect lion populations from potential criminal activity.

The extent to which wild lions are laundered as farmed lions is still unquantified, and the direct link between the trade in farmed lion parts and the poaching of wild lions is largely unknown [26,34,40]. This reveals a concerning knowledge gap with potentially detrimental consequences for lion populations across their range. This is of particular concern when considering the rising number of targeted poaching incidents of wild lions for their body parts across Africa, which is now recognised as a threat to lion conservation [22]. This criterion was not fully satisfied.

### 4.6. Conservation Implications of the Legal Captive Lion Industry in South Africa

In recent decades, CLB in South Africa has rapidly increased in terms of both scope and scale [13]. However, we found a lack of evidence-based information for a proper evaluation of its potential conservation benefits. Furthermore, we found some research that suggested that multiple criteria were not fully met, raising concerns that CLB could facilitate the demand for lion parts. Consequently, although we do not consider failure to meet the outlined criteria as an absolute dismissal of any conservation value, our review of the current relevant literature against the specified criteria framework (Table 2) indicates that there is reasonable cause to doubt that lion farming currently provides a sustainable supply side intervention to reduce pressure on wild lion populations.

Given the precarious status of lion populations on a global scale [22], our review suggests that the assumption that the CLB industry has a neutral or beneficial impact on wild lion populations should be approached by conservationists, policymakers, and government agencies with caution. Moreover, it is possible that similar lessons can also be learned from the parallel case of tiger farming, where the legal trade in parts and derivatives from captive tigers has failed to reduce pressure on wild tiger populations, despite protection as a CITES Appendix I listed species, which prohibits international commercial trade. More than 7000 tigers are bred in captivity across Asia to supply body parts and derivatives for the Asian market; however, wild tiger populations remain on the verge of extinction [59,83]. Concerns have also been raised that lion trade exacerbates the perceived availability and acceptability of tiger products, stimulating further demand [38]. The prediction that tiger populations would be decimated, causing consumers to resort to substitutions of lions and leopards, was already raised in the late 1990s [58,84]. The current market appears to follow suit, as indicated by the occurrence of tigers in commercial captive facilities in South Africa [17,81] and increased poaching and trade in a range of wild carnivore species [85,86]. The emergence of market supply and demand for the commercial use of wild felids should be subject to scrutiny, given the plethora of concerns raised about the impact on tiger and lion populations, as well as the animal welfare and public health concerns associated with all wildlife farming and trade activities [87,88].

The same concern applies to the emergence of commercial captive facilities outside South Africa. Currently, the South African government is on a trajectory to close the CLB industry through a consultative process that began in 2018. In 2020, a High-Level Panel of experts was appointed to review policies, practices, and management of captive breeding, keeping, and trade of lions. Their recommendations to terminate the commercial industry and all its associated commercial activities were approved by the Cabinet, based on the conclusions that CLB does not contribute to the conservation of wild lions, shows inherent animal welfare and public health concerns, poses concerns over the safety of workers and visiting public, and presents a threat to South Africa’s reputation as a responsible ecotourism destination, with associated political and economic risks [89,90]. These concerns were later corroborated by a Ministerial Task Team that was created to identify voluntary exit options from the CLB industry [13] and a Policy Position on the Conservation and Sustainable use of South Africa’s Biodiversity.

## 5. Limitations

Although we made extensive efforts to ensure that all relevant literature pertaining to lion farming was captured in our database, it is possible that some sources were missed in our search. To maintain the focus of our article on lion farming as a supply side intervention, we limited our search criteria to terms directly relevant to the commercial captive breeding of lions in South Africa. In doing so, we did not specifically include other lion range states in our search terms or terms related to trophy hunting and conservation of wild populations more broadly. However, we believe that our search provides a robust assessment of relevant information across these convergent issues. Furthermore, we limited the publications to the English language only.

We acknowledge that there are intersecting markets for the use and trade of lion body parts across Africa [21,68] and that lion populations do not exist in isolation. Therefore, we also collated information about lion populations outside South Africa that were relevant to captive lion breeding and trade which we have used in the summaries and discussion throughout our article. Future research that specifically includes all countries with wild lion populations would build on the findings presented here.

We adapted the evaluation framework by Tensen [1], similar to that used by Davies et al. [35], which is based on five pre-existing criteria originally derived from Biggs et al. [91]. While these criteria offer a useful conceptual basis for evaluating the quantity and quality of evidence across key areas, we found that complex interactions and trade-offs between criteria can make it difficult to assess the implications of violations for wild population exploitation [35]. Consequently, we propose that future evaluation frameworks should explicitly account for the interactions between criteria. Additionally, we did not critically assess the limitations of the data presented in each article across our dataset as part of our analysis. However, we reduced the likelihood of presenting misleading information by clearly distinguishing between peer-reviewed and grey literature sources and contextualising all the data reported. We do not consider failure to meet our criteria as a definitive hallmark to conclude that lion farming has a detrimental effect on wild populations. Rather, we believe that the framework is more likely to have value as an indicator of where knowledge gaps pertaining to the effects of the commercial lion industry lie and where caution or further attention to the potential impact of lion farms on wild populations is warranted.

## 6. Conclusions

Our review of the literature on CLB in South Africa suggests that there is currently inadequate evidence to definitively state that the industry has a positive impact on wild lion populations, either now or in the future. This level of uncertainty is, however, not uncommon in this kind of research, as is evident from the example of Allen et al. [92], who assert that the presently available evidence in support of positive dingo management is weak and signals caution, as well as Davis et al. on the bear bile industry in Vietnam [93]. While we do not suggest that our framework is conclusive in confirming that the CLB industry puts additional pressure on wild lion conservation, we draw attention to several “red flags” that indicate that the industry could have adverse effects on wild lion populations by facilitating the commercial demand for lions and their parts and derivatives. These red flags not only raise concerns but also highlight critical areas where further research is needed to better understand the potential implications of the industry. In particular, we recommend that research focus on confirmed consumer preferences, captive supply and demand interactions, economic comparisons between farmed and wild lion products, the genetics of captive populations, and the extent of illegal activity be prioritised, given the notable lack of information encountered during our review and the critical importance of these issues for informing lion conservation decision-making. Consequently, although wild lion populations in South Africa are currently considered stable, we highlight the risk that this additional pressure may pose, as well as the potential detrimental compounding effects on already vulnerable lion populations and other large felid species across their ranges [22]. Therefore, we stress the importance of monitoring and regulation to protect against criminal exploitation related to commercial captive facilities, particularly during the transition to terminating the industry in South Africa. Furthermore, we emphasise the need for caution in other countries, particularly lion range states, to heed South Africa’s position and refrain from facilitating the further emergence of commercial captive predator breeding and trade, particularly given the increased opportunity for exploitative wildlife trafficking between the African continent and Southeast Asia, for example, the expansion of the Belt and Road Initiative [94]. Our results generate urgent caution for CLB as a supply side approach to reduce pressure on wild populations while attempting to meet consumer demand for commercial use and trade of parts. We suggest that these insights may have important implications for the policy and governance of emerging commercial predator breeding operations in South Africa and globally.

## Figures and Tables

**Table 1:** Criteria framework for evaluating the species conservation impacts of commercial captive lion breeding in South Africa. Adapted from Tensen [1].

Criteria	Description of Relevant Info
Legally farmed lion parts provide a substitute for wild lion parts	1.1. Evidence of consumer preference for wild-caught or farmed lion parts and derivatives (consumer preference can be due to a number of factors, including taste, perceived value, perceived authenticity, perceived rarity, perceived strength, etc.) 1.2. Evidence of quality variation for wild-caught or farmed lion parts and derivatives
2. Farmed supply of lion parts and derivatives serves current market demand	2.1. Changes in demand for lion parts and derivatives 2.2. Changes in the quantity of traded lion parts and derivatives 2.3. Evidence of new markets emerging for the consumption or trade of lion parts and derivatives from farms 2.4. Breeding productivity rates on farms
3. Lion parts and derivatives from farmed populations are more cost-efficient than wild counterparts	3.1. Relative price of farmed and wild-sourced lion parts and derivatives for consumers 3.2. Relative price of farmed and wild-sourced lion parts and derivatives along the market chain 3.3. Relative profitability of farm facilities and sourcing from the wild (when factoring in the cost of maintaining high welfare standards) 3.4. Breeding productivity in relation to output required for profitability
4. Captive populations can be maintained without restocking from wild populations	4.1. Captive populations are entirely comprised of farmed lions, not wild individuals 4.2. Genetic variation can be maintained when breeding captive population only
5. Wild populations are sufficiently protected from criminal activity relating to lion farm facilities	5.1. Evidence of laundering wild-sourced lion parts and derivatives via farm facilities 5.2. Evidence of laundering parts and derivatives of other big cat species via lion farm facilities 5.3. Evidence of poaching lion parts and derivatives from farm facilities 5.4. Sufficient regulatory oversight with appropriate licences, permits, and an enforcement framework 5.5. Established techniques and capacity for distinguishing between captive-bred and wild-sourced lion parts and derivatives

**Table 2:** Summary of peer-reviewed literature sources with primary data and knowledge gaps for each of the five criteria used in the framework for evaluating the species conservation impacts of commercial lion farming, as adapted from Tensen [1].

Criteria	Number of Peer-Reviewed Articles Identified in Search	Peer-Reviewed Sources with Relevant Primary Data	Criteria Knowledge Gaps
Criterion 1: Legally farmed lion parts provide a substitute for wild lion parts	14	Four articles relating to consumer preference for wild versus farmed lions: [14,40,41,42].	Surveys among actual consumers are absent from the current literature; only surveys with the general public in consumer countries have been conducted.
Criterion 2: Farmed supply of lion parts and derivatives serves current market demand	51	Ten articles relating to the market supply and demand for lion products: [14,15,19,21,26,42,43,44,45,46].	There is currently no evidence to determine whether the supply of captive lions in South Africa satiates international demand for lion body parts in East and South-East Asia.
Criterion 3: Lion parts and derivatives from farmed populations are more cost efficient than wild counterparts	16	Three articles relating to the profitability of lion products: [19,43,44].	No direct comparison of price points or overall profitability between wild and captive-sourced lion products is available.
Criterion 4: Captive populations can be maintained without restocking from wild populations	14	Three articles relating to how the captive population is maintained: [19,47,48]. The same survey dataset was used for both [19,47].	No comprehensive genetic analysis of the captive population has been undertaken, and a reliably maintained studbook system is absent across the industry.
Criterion 5: Wild populations are sufficiently protected from criminal activity relating to lion farm facilities	42	Thirteen articles relating to laundering and poaching of wild lions: [14,15,16,19,21,26,44,49]. Relating to enforcement capacity and regulatory oversight: [16,46,50]. Relating to the capacity for distinguishing between captive-bred and wild-sourced lion parts, as well as between lions and other large felids: [51,52,53,54].	Much of the available data relating to this criterion is sporadic and anecdotal. Direct evidence of the link between the poaching of wild lions and the commercial captive industry is absent, the extent to which wild lions are laundered as farmed lions is unquantified, and the overall impact of criminal activity on wild lion populations relating to lion farm facilities is largely unknown.

## Appendix A

### Appendix A.1

**Table A1:** PRISMA 2020 Checklist.

Section and Topic	Item	Checklist Item	Location Where Item Is Reported
**TITLE**			
Title	1	Identify the report as a systematic review.	Abstract, Section 2.1
**ABSTRACT**			
Abstract	2	See the PRISMA 2020 for Abstracts checklist.	Abstract
**INTRODUCTION**			
Rationale	3	Describe the rationale for the review in the context of existing knowledge.	Section 2.1
Objectives	4	Provide an explicit statement of the objective(s) or question(s) the review addresses.	Section 1
**METHODS**			
Eligibility criteria	5	Specify the inclusion and exclusion criteria for the review and how studies were grouped for the syntheses.	Section 2.1
Information sources	6	Specify all databases, registers, websites, organisations, reference lists, and other sources searched or consulted to identify studies. Specify the date when each source was last searched or consulted.	Section 2.1
Search strategy	7	Present the full search strategies for all databases, registers, and websites, including any filters and limits used.	Section 2.1
Selection process	8	Specify the methods used to decide whether a study met the inclusion criteria of the review, including how many reviewers screened each record and each report retrieved, whether they worked independently, and, if applicable, details of automation tools used in the process.	Section 2.1
Data collection process	9	Specify the methods used to collect data from reports, including how many reviewers collected data from each report, whether they worked independently, any processes for obtaining or confirming data from study investigators, and, if applicable, details of automation tools used in the process.	Section 2.2
Data items	10a	List and define all outcomes for which data were sought. Specify whether all results that were compatible with each outcome domain in each study were sought (e.g., for all measures, time points, analyses), and if not, the methods used to decide which results to collect.	Section 2.2, Table 1
	10b	List and define all other variables for which data were sought (e.g., participant and intervention characteristics, funding sources). Describe any assumptions made about any missing or unclear information.	Section 2.2
Study risk of bias assessment	11	Specify the methods used to assess risk of bias in the included studies, including details of the tool(s) used, how many reviewers assessed each study, and whether they worked independently, and if applicable, details of automation tools used in the process.	Section 2.1 and Section 2.2. Table A2 and Table A3
Effect measures	12	Specify for each outcome the effect measure(s) (e.g., risk ratio, mean difference) used in the synthesis or presentation of results.	N/A as no quantitative data analysis performed
Synthesis methods	13a	Describe the processes used to decide which studies were eligible for each synthesis (e.g., tabulating the study intervention characteristics and comparing against the planned groups for each synthesis (item #5)).	N/A as no quantitative data analysis performed
	13b	Describe any methods required to prepare the data for presentation or synthesis, such as handling of missing summary statistics or data conversions.	N/A as no quantitative data analysis performed
	13c	Describe any methods used to tabulate or visually display the results of individual studies and syntheses.	N/A as no quantitative data analysis performed
	13d	Describe any methods used to synthesize results and provide a rationale for the choice(s). If meta-analysis was performed, describe the model(s), method(s) to identify the presence and extent of statistical heterogeneity, and software package(s) used.	N/A as no quantitative data analysis performed
	13e	Describe any methods used to explore possible causes of heterogeneity among study results (e.g., subgroup analysis, meta-regression).	N/A as no quantitative data analysis performed
	13f	Describe any sensitivity analyses conducted to assess the robustness of the synthesized results.	N/A as no quantitative data analysis performed
Reporting bias assessment	14	Describe any methods used to assess the risk of bias due to missing results in a synthesis (arising from reporting biases).	Section 2.1 and Section 2.2. Table A2 and Table A3
Certainty assessment	15	Describe any methods used to assess certainty (or confidence) in the body of evidence for an outcome.	Section 2.1 and Section 2.2. Table A2 and Table A3
**RESULTS**			
Study selection	16a	Describe the results of the search and selection process, from the number of records identified in the search to the number of studies included in the review, ideally using a flow diagram.	Section 3
	16b	Cite studies that might appear to meet the inclusion criteria, but which were excluded, and explain why they were excluded.	Section 2.1
Study characteristics	17	Cite each included study and present its characteristics.	Table 2, Table A2 and Table A3
Risk of bias in studies	18	Present assessments of risk of bias for each included study.	Table A2 and Table A3
Results of individual studies	19	For all outcomes, present, for each study: (a) summary statistics for each group (where appropriate) and (b) an effect estimate and its precision (e.g., confidence/credible interval), ideally using structured tables or plots.	Qualitative analysis of data presented in Table 2, Section 3 and Section 4.1, Section 4.2, Section 4.3, Section 4.4 and Section 4.5
Results of syntheses	20a	For each synthesis, briefly summarise the characteristics and risk of bias among contributing studies.	Section 2.1. Table A2 and Table A3
	20b	Present results of all statistical syntheses conducted. If meta-analysis was done, present for each the summary estimate and its precision (e.g., confidence/credible interval) and measures of statistical heterogeneity. If comparing groups, describe the direction of the effect.	N/A as no quantitative data analysis performed
	20c	Present results of all investigations of possible causes of heterogeneity among study results.	N/A as no quantitative data analysis performed
	20d	Present results of all sensitivity analyses conducted to assess the robustness of the synthesized results.	N/A as no quantitative data analysis performed
Reporting biases	21	Present assessments of risk of bias due to missing results (arising from reporting biases) for each synthesis assessed.	Section 2.1 and Section 5
Certainty of evidence	22	Present assessments of certainty (or confidence) in the body of evidence for each outcome assessed.	N/A as no quantitative data analysis performed
**DISCUSSION**			
Discussion	23a	Provide a general interpretation of the results in the context of other evidence.	Section 4
	23b	Discuss any limitations of the evidence included in the review.	Section 2.2 and Section 5
	23c	Discuss any limitations of the review processes used.	Section 2.2 and Section 5
	23d	Discuss implications of the results for practice, policy, and future research.	Section 6
**OTHER INFORMATION**			
Registration and protocol	24a	Provide registration information for the review, including register name and registration number, or state that the review was not registered.	Section 2.1
	24b	Indicate where the review protocol can be accessed, or state that a protocol was not prepared.	Section 2.1
	24c	Describe and explain any amendments to information provided at registration or in the protocol.	N/A, the review was not registered
Support	25	Describe sources of financial or non-financial support for the review, and the role of the funders or sponsors in the review.	Section Funding
Competing interests	26	Declare any competing interests of review authors.	Section Conflict of Interest
Availability of data, code and other materials	27	Report which of the following are publicly available and where they can be found: template data collection forms; data extracted from included studies; data used for all analyses; analytic code; any other materials used in the review.	Table A2 and Table A3

Source: Page, M.J., McKenzie, J.E., Bossuyt, P.M., Boutron, I., Hoffmann, T.C., Mulrow, C.D., et al. The PRISMA 2020 statement: an updated guideline for reporting systematic reviews. BMJ 2021;372:n71. doi: 10.1136/bmj.n71, [36]. This work is licensed under CC BY 4.0. To view a copy of this license, visit https://creativecommons.org/licenses/by/4.0/, (accessed on 14 April 2025).

### Appendix A.2. Literature Search Source Material Database

**Table A2:** Peer-reviewed literature sources.

Lead Author	Publication Year	Article Title	Journal	Impact Factor *	DHET Accredited **	Risk of Bias ***
Allen	2023	Why humans kill animals and why we cannot avoid it	Science of the Total Environment	8.6	Yes	Low
Bauer	2018	Lions in the modern arena of CITES	Conservation Letters	8.1	Yes	Low
Bauer	2022	Threat analysis for more effective lion conservation	Oryx	2.7	Yes	Low
Bauer	2015	Lion (*Panthera leo*) populations are declining rapidly across Africa, except in intensively managed areas	PNAS	10.8	Yes	Low
Becker	2022	Guidelines for evaluating the conservation value of African lion (*Panthera leo*) translocations	Frontiers in Conservation Science	1.9	Yes	Low
Bertola	2021	Genetic guidelines for translocations: Maintaining intraspecific diversity in the lion (*Panthera leo*)	Evolutionary Applications	4.2	Yes	Low
Blumenauer	2015	Changing Humanity: Fifteen Years of Progress in Animal Welfare and Protection	Animal Law Review	4	No	Low
Bodasing	2022	The decline of large carnivores in Africa and opportunities for change	Biological Conservation	6	Yes	Low
Braverman	2013	Conservation without nature: the trouble with in situ versus ex situ conservation	Geoforum	3.9	Yes	Low
Buk	2018	Conservation of severely fragmented populations: lessons from the transformation of uncoordinated reintroductions of cheetahs (Acinonyx jubatus) into a managed metapopulation with self-sustained growth	Biodiversity and Conservation	3.5	Yes	Low
Caro	2014	Conservation and behavior of Africa’s “Big Five”	Current Zoology	1.9	Yes	Low
Chorney	2022	Poor welfare indicators and husbandry practices lion “cub petting” facilities: evidence from public youtube videos	Animals	3	Yes	Low
Coals	2019	The Ethics of Human–Animal Relationships and Public Discourse: A Case Study of Lions Bred for Their Bones	Animals	3	Yes	Low
Coals	2022	Contemporary Cultural Trade of Lion Body Parts	Animals	3	Yes	Low
Coals	2021	DART mass spectrometry as a potential tool for the differentiation of captive-bred and wild lion bones	Biodiversity and Conservation	3.5	Yes	Low
Coals	2020	Commercially-driven lion part removal: What is the evidence from mortality records?	Global Ecology and Conservation	4.1	Yes	Low
Coals	2020	Preferences for lion and tiger bone wines amongst the urban public in China and Vietnam	Journal for Nature Conservation	2.5	Yes	Low
Coals	2019	Deep uncertainty, public reason, the conservation of biodiversity and the regulation of markets for lion skeletons	Sustainability	3.6	Yes	Low
Conrad	2012	Trade Bans: A Perfect Storm for Poaching?	Tropical Conservation Science	2.1	Yes	Low
Cooper	2013	Book chapter: conclusions and the way forward	Wildlife Forensic Investigation: Principles and Practice	2.4	Yes	Low
Cousins	2008	Exploring the Role of Private Wildlife Ranching as a Conservation Tool in South Africa: Stakeholder Perspectives	Ecology and Society	4.5	Yes	Low
Cousins	2010	The Challenge of Regulating Private Wildlife Ranches for Conservation in South Africa	Ecology and Society	4.5	Yes	Low
Cox	2012	Manipulating Resource Use by Goats With Predator Fecal Odors	Wildlife Society Bulletin	1.9	Yes	Low
Dalton	2018	A tale of the traded cat: development of a rapid real-time PCR diagnostic test to distinguish between lion and tiger bone	Conservation Genetics Resources	0.8	Yes	Low
Darkoh	2014	Okavango Delta—A Kalahari Oasis Under Environmental Threats	Journal of Biodiversity & Endangered Species	2.7	No	Low
D’Cruze	2020	Trading tactics: Time to rethink the global trade in wildlife	Animals	3	Yes	Low
D’Cruze	2015	Clouded in mystery: the global trade in clouded leopards	Biodiversity and Conservation	3.5	Yes	Low
D’Cruze	2016	A review of global trends in CITES live wildlife confiscations	Nature Conservation	2.5	Yes	Low
de Waal	2022	The unregulated nature of the commercial captive predator industry in South Africa: Insights gained using the PAIA process	Nature Conservation	2.5	Yes	Low
Dunston	2017	An assessment of African lion *Panthera leo* sociality via social network analysis: prerelease monitoring for an ex situ reintroduction program	Current Zoology	1.9	Yes	Low
Dutton	2013	Book chapter: Tackling unsustainable wildlife trade	Key Topics in Conservation Biology 2	N/A	Yes	Low
Everatt	2019	Evidence of a further emerging threat to lion conservation; targeted poaching for body parts	Biodiversity and Conservation	3.5	Yes	Low
Fletcher-Barnes	2021	Cuddle, kill, conserve: a posthuman analysis of the African lion within the South African wildlife security assemblage	International Journal of Sociology and Social Policy	1.9	Yes	Low
Fromentin	2023	Status, challenges and pathways to the sustainable use of wild species	Global Environmental Change	10.5	Yes	Low
Fukushima	2021	Challenges and perspectives on tackling illegal or unsustainable wildlife trade	Biological Conservation	6	Yes	Low
Gazendam	2023	A new approach to the vasectomy of African lions (*Panthera leo*)	Journal of the South African Veterinary Association	1.5	Yes	Low
Gratwicke	2008	Attitudes Toward Consumption and Conservation of Tigers in China	PLOS One	3.3	Yes	Low
Green	2020	African Lions and Zoonotic Diseases: Implications for Commercial Lion Farms in South Africa	Animals	3	Yes	Low
Green	2021	Ending Commercial Lion Farming in South Africa: A Gap Analysis Approach	Animals	3	Yes	Low
Green	2022	Wildlife Trade for Belief-Based Use: Insights From Traditional Healers in South Africa	Frontiers in Ecology and Evolution	3.4	Yes	Low
Green	2023	Taking stock of wildlife farming: A global perspective	Global Ecology and Conservation	4.1	Yes	Low
Green	2014	Surveys of lions *Panthera leo* in protected areas in Zimbabwe yield disturbing results: what is driving the population collapse?	Oryx	2.7	Yes	Low
Groom	2019	Animal welfare, social license, and wildlife use industries	Journal of Wildlife Management	2.4	Yes	Low
Hampton	2023	Poaching Forensics: Animal Victims in the Courtroom	Annual Review of Animal Biosciences	10.4	Yes	Low
Harper	2023	Welfare concerns associated with captive lions (*Panthera leo*) and the implications for commercial lion farms in South Africa	Animal Welfare	1.4	Yes	Low
Harvey	2020	Towards a cost-benefit analysis of South Africa’s captive predator breeding industry	Global Ecology and Conservation	4.1	Yes	Low
Heinrich	2022	The extent and nature of the commercial captive lion industry in the Free State province, South Africa	Nature Conservation	2.5	Yes	Low
Hiller	2021	How worldview and personal values can shape conservation conflict—The case of captive-bred lions	Biological Conservation	6	Yes	Low
Hinsley	2020	Building sustainability into the belt and road initiative’s traditional Chinese medicine trade	Nature Sustainability	30.2	Yes	Low
Hodgetts	2018	Improving the role of global conservation treaties in addressing contemporary threats to lions	Biodiversity and Conservation	3.5	Yes	Low
Holechek	2018	Wildlife Conservation on the Rangelands of Eastern and Southern Africa: Past, Present, and Future	Rangeland Ecology and Management	2.5	Yes	Low
Hughes	2023	Determining the sustainability of legal wildlife trade	Journal of Environmental Management	3.5	Yes	Low
Hunter	2012	Walking with lions: why there is no role for captive-origin lions *Panthera leo* in species restoration	Oryx	2.7	Yes	Low
Hutchinson	2020	Differentiating captive and wild African lion (*Panthera leo*) populations in South Africa, using stable carbon and nitrogen isotope analysis	Biodiversity and Conservation	3.5	Yes	Low
IUCN Assessment	2023	Lion *Panthera leo* has most recently been assessed for The IUCN Red List of Threatened Species in 2023	International Union for Conservation of Nature and Natural Resources	N/A	Yes	Low
Jaleel	2022	Letting the (Big) Cat(s) out of the Bag: A Case for Federal Prohibition of Exotic Big Cat Ownership in Pakistan	Journal of Animal and Environmental Law	2	No	Low
Jhala	2019	Asiatic Lion: Ecology, Economics, and Politics of Conservation	Frontiers in Ecology and Evolution	3.4	Yes	Low
Johanisova	2023	Assessing trophy hunting in South Africa by comparing hunting and exporting databases	Journal for Nature Conservation	2.5	Yes	Low
Kawase	2021	Contraceptive effect of a gonadotropin-releasing hormone vaccine on a captive female African Lion (*Panthera leo*): a case study	Journal of Veterinary Science	1.5	Yes	Low
Kettles	2009	Management of free-ranging lions on an enclosed game reserve: research article	South African Journal of Wildlife Research	0.3	No	Medium
Kurt	2020	Treatment of Pathological Fractures in Two Lion Cubs (*Panthera leo*) with Nutritional Secondary Hyperparathyroidsm	Kafkas Univ Vet Fak Derg	0.8	Yes	Low
Liew	2021	International socioeconomic inequality drives trade patterns in the global wildlife market	Science Advances	13.7	Yes	Low
Lindsey	2012	Possible relationships between the South African captive-bred lion hunting industry and the hunting and conservation of lions elsewhere in Africa: research article	South African Journal of Widlife Research	0.3	No	Medium
Lindsey	2013	The bushmeat trade in African savannas: Impacts, drivers, and possible solutions	Biological Conservation	6	Yes	Low
Lindsey	2017	The performance of African protected areas for lions and their prey	Biological Conservation	6	Yes	Low
Lindsey	2012	The Significance of African Lions for the Financial Viability of Trophy Hunting and the Maintenance of Wild Land	PLOS One	3.3	Yes	Low
Lindsey	2013	The Zambian Wildlife Ranching Industry: Scale, Associated Benefits, and Limitations Affecting Its Development	PLOS One	3.3	Yes	Low
Lindsey	2013	Determinants of Persistence and Tolerance of Carnivores on Namibian Ranches: Implications for Conservation on Southern African Private Lands	PLOS One	3.3	Yes	Low
Lindsey	2018	More than $1 billion needed annually to secure Africa’s protected areas with lions	Proceedings of the National Academy of Sciences	10.8	Yes	Low
Lunstrum	2020	What drives commercial poaching? From poverty to economic inequality	Biological Conservation	6	Yes	Low
Lunstrum	2017	Feed them to the lions: Conservation violence goes online	Geoforum	3.9	Yes	Low
MacDonald	2021	Trading Animal Lives: Ten Tricky Issues on the Road to Protecting Commodified Wild Animals	BioScience	9.7	Yes	Low
MacDonald	2019	Brushes with the law: A conservation scientist’s perspective on legal solutions and impediments from scottish wildcats to african lions	Journal of International Wildlife Law and Policy	0.9	Yes	Low
Maher	2017	Book chapter: International trade in animals and animal parts	The Palgrave International Handbook of Animal Abuse Studies	2.7	Yes	Low
Marnewick	2023	Biting the Hand that Feeds You: Attacks by Captive Carnivores Cause Deaths and Injuries in South Africa	African Journal of Wildlife Research	1.2	Yes	Low
Masse	2019	Anti-poaching’s politics of (in)visibility: Representing nature and conservation amidst a poaching crisis	Geoforum	3.9	Yes	Low
Miller	2023	Genetic diversity and origin of captive lion (*Panthera leo*) in South Africa: an assessment and comparison to wild populations	Conservation Genetics	2.2	Yes	Low
Miller	2014	Evaluation of Microsatellite Markers for Populations Studies and Forensic Identification of African Lions (*Panthera leo*)	Journal of Heredity	2.8	Yes	Low
Montgomery	2023	Predicting the consequences of subsistence poaching on the population persistence of a non-target species of conservation concern	Biological Conservation	6	Yes	Low
Mossaz	2015	Ecotourism contributions to conservation of African big cats	Journal for Nature Conservation	2.5	Yes	Low
Mweetwa	2018	Quantifying lion (*Panthera leo*) demographic response following a three-year moratorium on trophy hunting	PLOS One	3.3	Yes	Low
Nattrass	2021	Conservation and the Commodification of Wildlife in the Anthropocene: A Southern African History	South African Historical Journal	0.6	Yes	Low
Ndhlala	2011	Commercial herbal preparations in KwaZulu-Natal, South Africa: The urban face of traditional medicine	South African Journal of Botany	2.9	Yes	Low
Nelson	2013	Trophy hunting and lion conservation: a question of governance?	Oryx	2.7	Yes	Low
Ogada	2014	The power of poison: pesticide poisoning of Africa’s wildlife	Annals of the New York Academy of Sciences	6.3	Yes	Low
Ozioma	2019	Book chapter: Chapter 10—Herbal Medicines in African Traditional Medicine	Herbal Medicine	2.5	No	Low
Packer	2009	Sport Hunting, Predator Control and Conservation of Large Carnivores	PLOS One	3.3	Yes	Low
Peters	2023	Identifying priority locations to protect a wide-ranging endangered species	Biological Conservation	6	Yes	Low
Pitcher	2012	Lions, Tigers, and Emerging Markets: Africa’s Development Dilemmas	Current History	2.4	Yes	Low
Prisner-Levyne	2021	Trophy Hunting, Canned Hunting, Tiger Farming, and the Questionable Relevance of the Conservation Narrative Grounding International Wildlife Law	Journal of International Wildlife Law & Policy	0.9	Yes	Low
Riggio	2013	The size of savannah Africa: a lion’s (*Panthera leo*) view	Biodiversity and Conservation	3.5	Yes	Low
Rizzolo	2020	Wildlife Farms, Stigma and Harm	Animals	3	Yes	Low
Rizzolo	2021	Effects of legalization and wildlife farming on conservation	Global Ecology and Conservation	4.1	Yes	Low
Rizzolo	2023	Support for wildlife consumption bans and policies in China post-COVID-19	Oryx	2.7	Yes	Low
Schroeder	2018	Moving Targets: The ‘Canned’ Hunting of Captive-Bred Lions in South Africa	African Studies Review	1.8	Yes	Low
Seoraj-Pillai	2017	A Meta-Analysis of Human-Wildlife Conflict: South African and Global Perspectives	Sustainability	3.6	Yes	Low
Simon	2017	The competitive consumption and fetishism of wildlife trophies	Journal of Consumer Culture	2.7	Yes	Low
Slotow	2009	Book chapter: Chapter 3—Reintroduction Decisions Taken at the Incorrect Social Scale Devalue their Conservation Contribution: The African Lion in South Africa	Reintroduction of Top-Order Predators	N/A	Yes	Low
Tensen	2016	Under what circumstances can wildlife farming benefit species conservation?	Global Ecology and Conservation	4.1	Yes	Low
Toland	2020	Turning negatives into positives for pet trading and keeping: A review of positive lists	Animals	3	Yes	Low
Tricorache	2015	Book chapter: Chapter 14—Pets and Pelts: Understanding and Combating Poaching and Trafficking in Cheetahs	Cheetahs: Biology and Conservation	0.8	Yes	Low
Trinkel	2016	Book chapter: The Decline in the Lion Population in Africa and Possible Mitigation Measures	Problematic Wildlife	N/A	Yes	Low
Trouwborst	2017	International law and lions (*Panthera leo*): Understanding and improving the contribution of wildlife treaties to the conservation and sustainable use of an iconic carnivore	Nature Conservation	2.5	Yes	Low
‘t Sas-Rolfes	2019	Illegal Wildlife Trade: Scale, Processes, and Governance	Annual Review of Environment and Resources	18.9	Yes	Low
Turner	2020	Lion Conservation and the Lion Bone Trade in South Africa: On CITES, Shifting Paradigms, “Sustainable Use” and Rehabilitation	Oriental Anthropologist	2.8	Yes	Low
Uddin	2023	Laundered alive? The transnational trade in wild felids through Bangladesh	Global Ecology and Conservation	4.1	Yes	Low
Vaciano	2022	Post-Mortem Dental Profile as a Powerful Tool in Animal Forensic Investigations—A Review	Animals	3	Yes	Low
Van der Merwe	2018	The economic significance of lion breeding operations in the South African Wildlife Industry	International Journal of Biodiversity and Conservation	3.5	No	Low
Van der Weyde	2018	Multi-species occupancy modelling of a carnivore guild in wildlife management areas in the Kalahari	Biological Conservation	6	Yes	Low
Van der Weyde	2021	Collaboration for conservation: Assessing countrywide carnivore occupancy dynamics from sparse data	Diversity and Distributions	4.1	Yes	Low
van Hoven	2015	Book chapter: Private Game Reserves in Southern Africa	Institutional Arrangements for Conservation, Development and Tourism in Eastern and Southern Africa	N/A	Yes	Low
van Uhm	2019	Book chapter: Chinese wildlife trafficking networks along the Silk Road (pp. 114–133)	Organized crime and corruption across borders. Routledge.	N/A	Yes	Low
van Uhm	2016	Book: The Illegal Wildlife Trade: Inside the World of Poachers, Smugglers and Traders	The Illegal Wildlife Trade: Inside the World of Poachers, Smugglers and Traders	N/A	Yes	Low
White	2021	How China’s Wildlife Trade Legislation Permits Commercial Trade in Protected Wild Animal Species	The China Quarterly	2.9	Yes	Low
Whiting	2012	Book chapter: Animals Traded for Traditional Medicine at the Faraday Market in South Africa: Species Diversity and Conservation Implications	Animals in Traditional Folk Medicine	1	Yes	Low
Williams	2015	‘Skullduggery’: Lions Align and Their Mandibles Rock!	PLOS One	3.3	Yes	Low
Williams	2019	Born captive: A survey of the lion breeding, keeping and hunting industries in South Africa	PLOS One	3.3	Yes	Low
Williams	2021	Monitoring compliance of CITES lion bone exports from South Africa	PLOS One	3.3	Yes	Low
Williams	2017a	A roaring trade? The legal trade in *Panthera leo* bones from Africa to East-Southeast Asia	PLOS One	3.3	Yes	Low
Williams	2017b	Questionnaire survey of the pan-African trade in lion body parts	PLOS One	3.3	Yes	Low
Wilson	2023	Behaviour and welfare of African lion (*Panthera leo*) cubs used in contact wildlife tourism	Animal Welfare	1.4	Yes	Low
Wilson	2019	Animal Law in South Africa: ‘Until the Lions Have Their Own Lawyers, the Law Will Continue to Protect the Hunter’	Derecho Animal (Forum of Animal Law Studies)	-	Yes	Low
Wyatt	2021	Book chapter: Reflecting on Wildlife Trafficking	Wildlife Trafficking	N/A	Yes	Low
Yamaguchi	2009	Brain size of the lion (*Panthera leo*) and the tiger (*P. tigris*): implications for intrageneric phylogeny, intraspecific differences and the effects of captivity	Biological Journal of the Linnean Society	1.8	Yes	Low
Zhao	2020	Metacoupled Tourism and Wildlife Translocations Affect Synergies and Trade-Offs among Sustainable Development Goals across Spillover Systems	Sustainability	3.6	Yes	Low
Zhu	2023	Perceptions of COVID-19 origins and China’s wildlife policy reforms	Global Ecology and Conservation	4.1	Yes	Low

* 5-year impact factor. If not available, the 2-year impact factor was used. ** South African Department of Higher Education and Training Accredited Journals 2025-26, based on IBSS, Scopus, Norwergian, Web of Science, ScieLO SA, and DOAJ indices. *** Risk of Bias medium if journal is not DHET accredited and IF below 1.5, otherwise the risk of bias is low.

**Table A3:** Grey literature sources.

Lead Author	Publication Year	Document Title	Publication Type	Risk of Bias	Risk of Bias Notes
Abell	2013	A Framework for the Ex Situ Reintroduction of the African Lion (*Panthera leo*)	Open Science repository	Medium	As a not peer-reviewed article, there is a risk of methodological flaws and lack of credibility in the findings.
Booyens	2021	The introduction of captive bred African lions (*Panthera leo*) to a private wildlife reserve in the Limpopo Province	Doctoral Research	Medium	As a doctoral thesis, it may lack peer review but provides valuable research-based insights.
Born Free	2018	Cash Before Conservation: An overview of the breeding of lions for hunting and bone trade	Report by Born Free	Medium	As an advocacy organization, they may have a strong position against the lion industry, potentially introducing bias.
Centre for Environmental Rights & Endangered Wildlife Trust	2019	Legal and practical regulation of the welfare of wild animals in South Africa: Principles for law reform	Report by Centre for Environmental Rights & Endangered Wildlife Trust	Medium	These organizations advocate for stronger wildlife protection laws, which may influence the conclusions of the report.
Coleman	2019	Lion bone industry grows in SA’s legislative vacuum	Agricultural magazine	Medium	Could have a potential bias towards promoting or downplaying issues regarding the lion bone trade.
Court case	2009	National Council of the Society for Prevention of Cruelty to Animals vs Minister of Environmental Affairs, Director-General, Department of Environmental Affairs and South African Predators Association, Case No. 86515/2017	Official court document	Low	Official court documents are based on legal findings and should be unbiased.
Department of Forestry, Fisheries, and the Environment	2021	Draft Policy Position on the conservation and sustainable use of elephant, lion, leopard and rhinoceros	Government white paper	Medium	As a government publication, the document could reflect the interests and policy priorities of the South African government.
Duffy	2013	Poverty, Poaching and Trafficking: What are the links?	Report comissioned by the UK Department for International Development	Medium	As a government-commissioned report, it might present data and conclusions aligning with the policy interests of the commissioning body.
Els	2016	Determining the economic significance of the lion industry in the private wildlife tourism sector	Master’s Thesis	Medium	As a thesis, it may lack the external scrutiny that peer-reviewed publications receive, although academic rigor is usually maintained.
EMS Foundation and Ban Animal Trading	2018	The extinction business: South Africa’s ‘lion’ bone trade.	Report by EMS Foundation and Ban Animal Trading	Medium-High	As an advocacy organization, they may have a strong position against the lion industry, potentially introducing bias.
Environmental Investigation Agency	2017	The lion’s share: South Africa’s trade exacerbates demand for tiger parts and derivative.	Report by Environmental Investigation Agency	Medium	The EIA is a respected NGO, but its advocacy position against wildlife trafficking could influence the framing of the issue.
Four Paws	2022	Year of the tiger? Big cat farming in South Africa: the need for international action	Report by Four Paws	Medium	Four Paws is an animal welfare organization, and their position may influence how the issue is framed.
Funston	2015	Biodiversity Management Plan for African Lion (*Panthera leo*)	Government report by Department of Environmental Affairs	Low	As a government document, it may reflect policy priorities, but it’s based on scientific management recommendations by scientists from Panthera
Harvey	2020	Working Paper: The Economics of Captive Predator Breeding in South Africa	Report by South African Institute of International Affairs	Medium	SAIIA is an independent think tank. As a working paper, it may still reflect early-stage research, and the publisher’s agenda could influence its framing.
Hiller	2022	Understanding South Africa’s captive lion sector and the trade in captive lions (*Panthera leo*)	Report by Endangered Wildlife Trust	Medium	As an advocacy organization, EWT’s stance may influence how the topic is framed.
Macdonald	2016	Report on lion conservation with particular respect to the issue of trophy hunting	Report by University of Oxford, WildCru	Low	As a report from a respected academic institution, it is likely to have undergone rigorous peer review.
Mandy	2021	Why conservation fails: uncovering the wicked political nature of Southern Africa’s fight against wildlife extinction	Master’s Thesis	Medium	As a Master’s thesis, it may lack the external scrutiny that peer-reviewed publications receive, although academic rigor is usually maintained.
Marnewick	2015	Conservation biology of cheetahs *Acinonyx jubatus* (Schreber, 1775) and African wild dogs *Lycaon pictus* (Temminck, 1820) in South Africa	PhD Thesis, University of Pretoria	Medium	As a doctoral thesis, it may lack peer review but provides valuable research-based insights.
National Assembly Question	2019	Question No 410 (NW1382E) (2019) to the Minister of Environment, Forestry and Fisheries on predator-breeding farms, Question No. 7 of 2019	National Assembly Question	Low	As a formal parliamentary question, the document should be factual and neutral.
National Assembly Question	2020	Question No 1994 (NW2555E) (2020) to the Minister of Environment, Forestry and Fisheries on lion bone export quota considerations, Question No. 34 of 2020	National Assembly Question	Low	As a formal parliamentary question, the document should be factual and neutral.
National Council of Societies for the Prevention of Cruelty to Animals	2017	Overview of inspection to lion breeding farms by the National Wildlife Protection Unit Inspectorate during the period March 2016–June 2017	Report by NSPCA	Medium	As a strong advocate for animal welfare, the NSPCA’s report may be presented with a particular advocacy lens.
National Geographic	2019	More than 100 neglected lions found in a South African breeding facility	National Geographic Magazine	Low	National Geographic is a reputable publication with a history of balanced reporting on environmental issues.
National Geographic	2019	Exclusive: Inside a controversial South African lion farm	National Geographic Magazine	Low	National Geographic is a reputable publication with a history of balanced reporting on environmental issues.
Oswell	2010	The Big Cat Trade in Myanmar and Thailand	Report by TRAFFIC	Low	TRAFFIC is a reputable organization that works on wildlife trade monitoring, so the risk of bias is relatively low.
Parliamentary meeting	2018	Committee Report on Colloquium on Captive Lion Breeding for Hunting in SA 21 & 22 August 2018 ATC181108: Report of the Portfolio Committee on Environmental Affairs on the Colloquium on Captive Lion Breeding for Hunting in South Africa: harming or promoting the conservation image of the country, held on 21 and 22 august 2018, dated 8 November 2018	Government report	Medium	As a government document, it may reflect policy positions or the interests of the governing parties.
Safari Club International	2019	Anti-Hunting Propaganda Ignores Reality	Industry report	High	Safari Club International is a hunting advocacy organization, and this report is likely to present a biased perspective on the issue.
Schoen	2022	Poaching Ourselves to Death: the Illegal Wildlife Trade	Blog	Medium	Blogs may lack peer review and can have personal or organizational biases.
Smithsonian	2019	108 neglected lions found on South African breeding farm	Smithsonian Magazine	Low	The Smithsonian is a respected institution with a commitment to factual reporting.
The Conversation	2019	Lion and tiger farming may be inhumane, but we don’t know if it increases poaching	The Conversation	Low	As an independent news source, typically publishing content from academic and research experts, it provides a balanced perspective.
The Revelator	2019	South Africa’s Fallen Pride: How Law and Government Fail to Protect Lions	The Revelator	Medium	As an independent environmental news outlet, it may provide an objective view, though its tone could reflect a particular angle.
Tigere	2020	A critical discussion of the legality of South Africa’s lion bone trade	Master’s Thesis	Medium	As a Master’s thesis, it may lack the external scrutiny that peer-reviewed publications receive, although academic rigor is usually maintained.
TRAFFIC	2015	Bones of contention: As assessment of the South African trade in African Lion (*Panthera leo*) bones and other body parts	Report by TRAFFIC	Low	TRAFFIC is a reputable organization that works on wildlife trade monitoring, so the risk of bias is relatively low.
TRAFFIC	2018	The Legal and Illegal Trade in African Lions	Report by TRAFFIC	Low	TRAFFIC is a reputable organization that works on wildlife trade monitoring, so the risk of bias is relatively low.
‘t Sas-Rolfes	2023	A conflict of visions: Ideas shaping wildlife trade policy toward African megafauna	People and Nature	Low	Pre-publication version of the peer-reviewed article.
Wiersema	2016	Incomplete Bans and Uncertain Markets in Wildlife Trade	University of Pennsylvania Asian Law Review	Low	As an article from a respected academic institution, it is likely to have undergone rigorous peer review.
World Animal Protection	2019	Trading Cruelty—how captive big cat farming fuels the traditional Asian medicine industry	Report by World Animal Protection	Medium	As an advocacy organization, it may present the issue from a specific standpoint.
Yale Environment 360	2018	The Ongoing Disgrace of South Africa’s Captive-Bred Lion Trade	University blog	Low	Yale Environment 360 is an authoritative platform for environmental issues and provides research-driven, well-informed perspectives.
